# Limb Salvage After Deep Tissue Infection Associated with Hydroxyurea Therapy and Recommendations for a Follow-Up Protocol in Patients Treated with Hydroxyurea

**DOI:** 10.3390/jcm14248637

**Published:** 2025-12-05

**Authors:** Éva Badak, Edina Bodnár, László Virág, Éva Remenyik, Éva Szabó

**Affiliations:** 1 Department of Dermatology, MTA Centre of Excellence, Faculty of Medicine, University of Debrecen, 4032 Debrecen, Hungary; badak.eva92@gmail.com (É.B.); edibodnar78@gmail.com (E.B.); remenyik@med.unideb.hu (É.R.); 2 General Practice, 7039 Németkér, Hungary; 3 Outpatient Centre, 4200 Hajdúszoboszló, Hungary; 4 Department of Medical Chemistry, MTA Centre of Excellence, Faculty of Medicine, University of Debrecen, 4032 Debrecen, Hungary; lvirag@med.unideb.hu; 5 HUN-REN-DE Cell Biology and Signaling Research Group, University of Debrecen, 4032 Debrecen, Hungary

**Keywords:** hydroxyurea, skin ulcer, osteomyelitis, complex treatment, follow up protocol

## Abstract

Hydroxyurea (HU) is a cytostatic drug used in oncotherapy. The drug has inhibitory effects on bone marrow and epithelial cells and causes minor side effects in the skin, including nail deformity, alopecia, and hyperpigmentation, and severe side effects, including skin tumors and nonhealing ulcers. Herein, we report the case of a patient who received HU therapy for polycythemia vera. The patient had type 2 diabetes and atherosclerosis. Onychodystrophy and a non-healing ulcer developed. Severe deep tissue infection and osteomyelitis, rare complications of a HU-related ulcer, were also diagnosed later. Diabetes and atherosclerosis made the condition more severe. Complex systemic and local therapy led to complete healing of the ulcer and osteomyelitis. Based on the literature and our own experience, a care protocol was proposed for the dermatological follow-up of patients under HU treatment. This recommendation may be particularly useful in the treatment of patients treated with hydroxyurea who suffer from atherosclerosis, diabetes, or leg ulcers and are therefore at increased risk of severe skin and deep tissue infections.

## 1. Introduction

Hydroxyurea (HU), also known as hydroxycarbamide, is an effective treatment for hematological disorders, including myeloproliferative diseases such as polycythemia vera and essential thrombocythemia [1,2]. HU reduces the synthesis of deoxyribonucleotides by inhibiting ribonucleotide reductase [2,3]. Consequently, DNA synthesis and cell division are inhibited. HU halts the cell cycle at the G1/S phase by inhibiting the M2 subunit of ribonucleotide reductase. Keeping cells in the G1 phase interferes with DNA repair and consequently sensitizes cells to radiation-induced damage [3]. Bone marrow is the primary site of HU-induced cytostasis, but HU also inhibits epithelial cell proliferation [4]. Therefore, side effects often develop in the skin of patients with hematological diseases treated with HU. The skin-related side effects include photosensitivity (acute sunburn, dermatomyositis-like symptoms, skin photoaging, photocarcinogenesis), alopecia, dry skin, and nail deformities (fragmentation and melanonychia) [5,6,7]. As a manifestation of photocarcinogenesis, squamous cell carcinoma is a serious side effect of HU treatment [7,8]. Therapy-resistant skin ulcers can also form as a side effect of HU [9,10,11,12,13]. These wounds can be treated in a number of different ways, including polyabsorbent polyacrylate fibers [14] and hyperbaric oxygen [15]. Nail fragmentation and dry skin can lead to infection in immunocompromised patients, and paronychia may develop. HU is often the first-line treatment for polycythemia vera. If the treatment is ineffective or the patient does not tolerate HU, then JAK inhibitors can be administered [16].

In the present case, a chronic wound led to the development of skin necrosis and osteomyelitis. These severe complications were triggered by a nail ablation, which was performed due to nailbed inflammation in a patient with polycythemia vera. After applying complex systemic and local conservative and surgical treatment for several months, modifying the hematological therapy, the lesion healed. Details of the successful therapy and the importance of an interdisciplinary approach are provided.

## 2. Case Presentation

A 56-year-old male patient was diagnosed with polycythemia vera 14 years before the appearance of dermatological side effects. When he first came to our clinic, he was taking HU (2 × 500 mg/day) and acetylsalicylic acid (100 mg/day p.o.). He was also taking oral antidiabetics (glikidon, 2 × 30 mg/day) for a year to treat type 2 diabetes. Half a year before admission to the dermatology unit for partial onycholysis, paronychia developed on his first right toe, and nail ablation was performed in the surgery department. After surgery, the wound did not heal for several months. Therefore, an angiography examination was recommended by the surgeon. The angiography did not show stenosis in the right lower limb, but the Doppler ultrasound revealed weakened monophasic flow in the back of the leg. Conservative intravenous antithrombotic vasodilator therapy (prostacycline 60 µg/day i.v. for 1 week; pentoxifylline 100 mg/day i.v. followed by 2 × 400 mg/day p.o.) was started, and an oral thrombocyte aggregation-inhibitor (acetylsalicylic acid 100 mg/day p.o. continuously) and a vasodilator (phosphodiesterase inhibitor, cilostazol 2 × 50 mg/day continuously) were prescribed.

**Figure 1:**
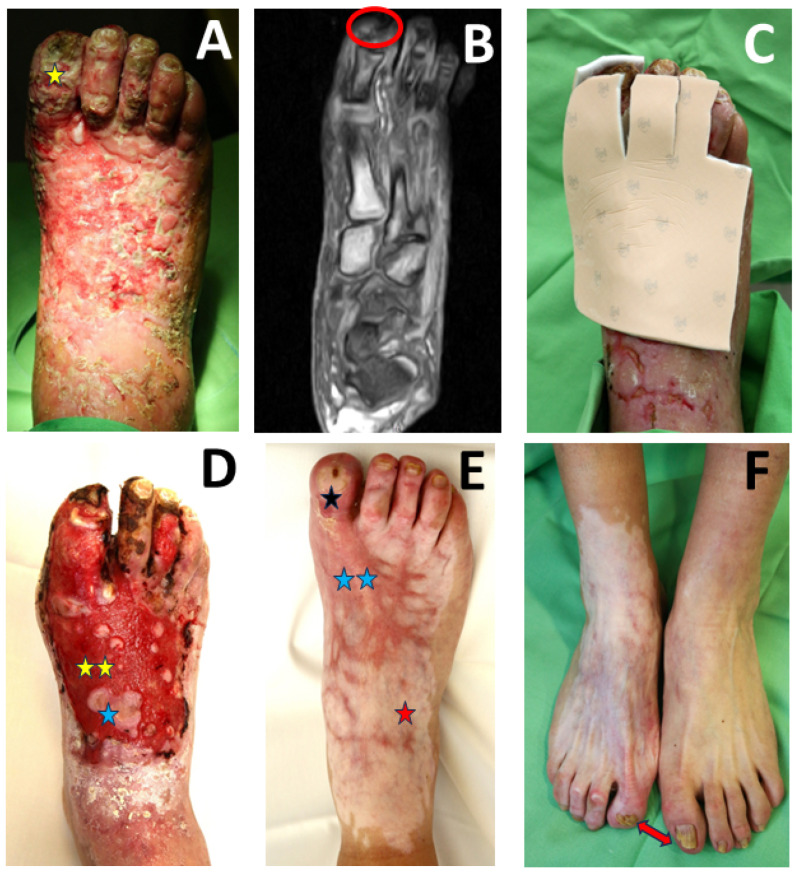
(**A**) Status at admission included necrosis in the nail ablation area on the first toe (yellow star symbol), on the dorsal surface of the foot, and on the II–IV toes of the right leg. (**B**) Magnetic resonance imaging of the right foot showed typical symptoms of osteomyelitis, bone marrow edema, and blurred bone contours in the distal part of the distal phalanx of the first toe (red circle). (**C**) After necrectomy, wound healing was enhanced with conservative treatment with a silver-containing bandage. (**D**) Granulation (two yellow stars) and epithelization (blue star symbol) stage of wound healing. (**E**) The ulcer and the deep tissue infection healed in 6 months. (**F**) The patient was asymptomatic for 18 months after the wound healed. The right first toe was shorter because the distal part of the distal phalanx was resorbed due to osteomyelitis. The inflammation resolved, and only a scar (two blue stars), hypopigmentation (red star symbol), and nail aberrations (black star symbol) remained. The red arrow indicates the shortened first right toe due to bone resorption.

The patient was referred to our department due to the non-healing ulcer and progression of the infection. Necrosis was detected at the nail ablation area on the first toe and on the dorsal surface of the foot and the II–IV toes (Figure 1A). The skin surrounding the ulcer was dry, scaly, slightly hyperemic, and infiltrated. Repeated color Doppler examination of the arterial trunks of the right lower limb showed regular flow conditions. However, Doppler examination of the peroneal area showed weak flow. The patient’s noteworthy laboratory results included WBC (25.1 G/L, ref: 4.5–10.8), platelet count (464 G/L, ref: 150–400), CRP (49.1 mg/L, ref: <5.2), limited renal function (eGFR: 27 mL/min/1.73 m^2^, ref: >90), and serum glucose levels close to the reference value (6.4 mmol/L, ref: 3.0–6.0). The foot and toes were X-rayed because of the severe infection and necrosis. The X-rays showed osteomyelitis in the distal phalanx area of toe I. This finding was confirmed by magnetic resonance (MR) (Figure 1B). *Enterococcus faecalis* and *Staphylococcus hemolyticus* were identified in bacteriological cultures of the wound.

After an orthopedic examination, conservative treatment for the bone involvement was recommended. Biopsies were collected from the base and the edge of the wound. The differential diagnoses included pyoderma gangrenosum, vasculitis, calciphylaxis, and exulcerated tumor. Histology revealed inflammation around the vessel walls in the connective tissue, consisting of lymphocytes without special alterations. Malignancy was excluded. Based on consultations with an infectologist and a nephrologist, the combination antibiotic therapy, consisting of glycopeptide (vancomycin, 500 mg/day) and a third-generation cephalosporine (ceftriaxone, 2 g/day), was administered for 14 days. The renal function improved, but the liver function deteriorated. Therefore, the antibiotics were changed to an aminoglycoside (gentamycin, 240 mg/day) and teicoplanin (400 mg/day) for 28 days. Local treatment was also initiated. Necrectomy and debridement were performed, and healing was enhanced using a silver-containing foam bandage (Figure 1C).

A hematology consultation was required due to the severe infection and large non-healing ulcer. Modification of the HU dose or a change to another drug was recommended. The patient did not consent to the IntronA treatment recommended by the hematologist, so the dose of HU was decreased from 2 × 500 mg/day to 500 mg/every other day. A previous genetic study of the patient confirmed a JAK2 mutation; therefore, a JAK inhibitor (2 × 5 mg/day ruxolitinib) was also started. The inflammation decreased, and systemic antibiotics were changed to oral oxazolidinone (linezolid, 2 × 600 mg/day). Based on the protocol recommended by the infectologist, the linezolid antibiotic treatment was continued for 24 weeks due to the osteomyelitis. The combination of antibiotic administration, local therapy, and modification of the hematological treatment caused wound granulation and resolution of the inflammation, and epithelization began (Figure 1D). The patient was discharged with a healing wound and a hematologically stable condition. The local and systemic treatment was continued at home with weekly follow-up. The wound healed after 6 months (Figure 1E). A follow-up X-ray examination showed partial resorption of the distal phalanx of the right first toenail without signs of osteomyelitis. The patient’s underlying hematological disease was balanced. Key laboratory findings at discharge were WBC 11.5 G/L (reference 4.5–10.8 G/L), platelet count 448 G/L (reference 150–400 G/L), CRP 11.4 mg/L (reference < 5.2 mg/L) and eGFR 27 mL/min/1.73 m^2^. The patient has been asymptomatic for 18 months after his wound healed; only hypopigmentation and nail aberration remain (Figure 1F).

## 3. Discussion

### 3.1. Skin Lesions Induced by Hydroxyurea

HU has been successfully used in tumor therapy, especially hematologic diseases, since 1960 [1]. However, long-term use of this drug can cause a wide array of side effects. As with all cytostatic treatments, general side effects occur, including fatigue, headache, and gastrointestinal symptoms. Mild skin symptoms also frequently develop, including dry skin, melanonychia (i.e., the brownish discoloration of the nails), dermatomyositis-like symptoms, alopecia, and hyperpigmentation, sun-damaged skin, and atrophy due to its photosensitizing effects [5,6]. Squamous cell carcinoma on sun-damaged skin may develop as a serious side effect of HU [8].

### 3.2. Non-Healing Ulcer, Deep Tissue Involvement Associated with Hydroxyurea Therapy

The development of therapy-resistant ulcers is a severe side effect of HU reported in many publications [9,10,11,12,13,17]. Stahl and Silber first reported leg ulcers as a side effect of HU in 1985 [18]. In addition to ulcers, Stahl and Silber described vasculitis and collagen degeneration in small veins [18]. Other publications reported perivascular lymphocytic infiltration without vasculitis and thickening of the vessel wall in the dermis induced by HU. Patients felt pain in the scar during transfusion of thrombocytes after complete healing of the ulcer, possibly due to ischemia development after intravascular thrombosis [19]. HU-associated ulcers are usually localized to the ankle area. Of note, trauma is also more common at this location.

HU causes skin toxicity through basal keratinocyte damage, skin atrophy, and altered collagen synthesis [9,20]. HU elicits cytostatic effects in both bone marrow cells and stem cells of the epidermis and endothelial cells. The drug’s toxic effects accumulate as the repair mechanisms are unable to keep up with the enhanced DNA damage [3]. According to several large-scale studies, the ulcers heal spontaneously after cessation of HU therapy or skin transplantation [9,10]. HU-induced ulcers are influenced by the anti-angiogenic effects of the drug [21]. In one study of 41 cases of ulcers after HU therapy [12], the histological examinations showed mainly epidermal atrophy, dermal fibrosis, and scar tissue without vascular lesions. In one case, occlusion of the small veins, similar to livedo vasculitis, was observed; however, immunofluorescent staining for vasculitis markers was negative. In another case, fibrinoid deposits with red blood cell extravasation and leukocytoclastic vasculitis were detected in the dermis. These ulcers occurred 5 years after starting HU therapy, suggesting that ulcers develop as a result of cumulative HU toxicity. The contribution of a vascular component could not be confirmed in that study [12]. Boyd et al. [22] proposed that ulcers develop due to the direct cytotoxic effect of HU. Moreover, in a cohort of 152 patients, including patients with myeloproliferative diseases, HU treatment was administered for 8.13 years, and the frequency of side effects was 6.5%. The side effects regressed after cessation of HU treatment [23]. Another study described the rare localization of a HU-related ulcer on the foot (above the metatarsophalangeal joint) of a patient treated for polycythemia vera; osteomyelitis and joint infection also developed. The patient’s ulcer had no arterial or neurological origin. The wound healed in 3 months after modifying the hematological therapy and surgical treatment [24].

In our patient with polycythemia vera and a 14-year-long treatment history with HU, the symptoms started with onychodystrophy and paronychia on the right first toe. The nail symptoms were also probably induced by HU. After nail ablation, a non-healing wound developed, accompanied by severe skin inflammation, skin/deep tissue necrosis, and osteomyelitis. Type 2 diabetes and atherosclerosis may have contributed to the severe condition. The diabetic environment promotes cellular aging [25], and diabetes contributes to arterial stiffness [26]. Impaired wound healing results from multiple factors in diabetes, including poor circulation, nerve damage, inflammation, changes in tissue structure, microbiome disruptions, and altered cell migration and proliferation [27]. Skin and soft tissue infections are common in diabetics, usually resulting from impaired skin barrier, trauma, or ischemic wounds [28]. Due to the skin and soft tissue infections, conservative therapy was recommended by orthopedics. (Due to the extensive necrotic ulcer, the orthopedic consultation did not recommend bone debridement.) Long-term antibiotic therapy and adequate local and modified hematological therapy resulted in complete wound healing.

Osteomyelitis is a rare complication of HU treatment; we found only one study reporting HU-associated osteomyelitis [24]. In that case report, an ulcer was followed by osteomyelitis of the metatarsophalangeal joint in a patient with polycytemia vera on HU therapy; surgery (amputation) was necessary in that case.

**Figure 2:**
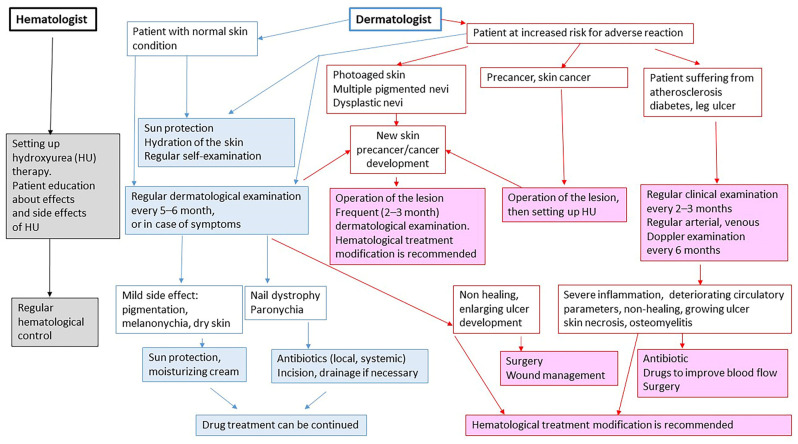
Follow-up protocol for patients treated with hydroxyurea (black frame with gray background: hematologist’s task when setting up HU treatment; blue frame: recommendations for dermatologists in case of mild side effects or minor skin conditions; blue border and blue background: treatment recommendations for managing mild side effects of HU; red box: clinical symptoms that pose a high risk of developing side effects of HU treatment; red frame with pink background: tasks in the event of serious side effects or complications arising from HU treatment).

Our case highlights the importance of close follow-up in patients treated with HU. A care protocol was developed for the dermatological follow-up of patients being treated with HU, based on the literature and our own experience (Figure 2). Patients with photo-damaged skin, dysplastic nevus, or a large number of pigmented nevi are at increased risk of developing skin malignancies. For patients with normal skin, we recommend sun protection, regular self-examination, and regular dermatological examination every 5–6 months or in case of symptoms. When mild side effects (pigmentation, melanonychia, dry skin) develop, sun protection and moisturizing cream are recommended, and HU treatment can be continued. When nail dystrophy and paronychia develop, antibiotics and incision are recommended, and drainage may be necessary, but HU treatment can be continued. Patients with photo-damaged skin, dysplastic nevi, or multiple pigmented nevi have an increased risk of skin malignancies. Therefore, they have an increased risk of adverse reactions to HU. More frequent (2–3 months) dermatological examination is necessary in these patients. In patients with precancerous (e.g., actinic keratosis) or malignant skin tumors, the lesion should be treated first, and HU treatment should be initiated after treatment of the lesion. Furthermore, patients suffering from atherosclerosis, diabetes, or leg ulcers have a higher risk of severe skin and deep tissue infections. Therefore, regular clinical (every 2–3 months) and Doppler (every 6 months) examinations are necessary in these patients. If the wound gets worse during HU treatment, the hematological therapy should be modified. If onychodystrophy, paronychia, or periulcer inflammation develops, the HU dose should be reduced, or the hematological therapy should be modified to avoid deep tissue infection (Figure 2).

The limitations of our case report and our recommendations based on it are that it is based on a single case and that the patient was not followed up with long-term imaging after complete recovery. However, we considered the case worthy of presentation due to its rarity and because the proposed protocol can prevent such serious complications.

## 4. Conclusions

HU is effective in treating hematological diseases. HU inhibits the proliferation of both bone marrow cells and cells of the epidermis. As a result, several skin side effects can develop in patients receiving long-term HU treatment. In addition to mild side effects such as dry skin, skin atrophy, hyperpigmentation, and alopecia, severe symptoms such as squamous cell carcinoma and non-healing ulcers can develop. In rare cases, deep tissue infections can develop. We present the case of a patient with polycythemia vera with diabetes and arteriosclerosis treated with HU. A necrotic ulcer and osteomyelitis developed, and the patient was successfully treated with conservative treatment, based on antibiotic treatment, local therapy, and modification of the hematological drug. Only one case of the development of an ulcer and osteomyelitis in a patient with polycythemia vera treated with HU has been published, but that patient required amputation [24].

Hematologists should be aware of the side effects of HU and raise awareness about the side effects with patients. Early detection of side effects helps prevent the development of more severe symptoms [8,24]. Nail bed inflammation and ulcers can progress to deep tissue infection, skin necrosis, and osteomyelitis. For efficient management of such scenarios, specialists in related fields (dermatology, surgery, hematology, and infectology) should be involved in the clinical decision-making. Modification of hematological treatment protocols (e.g., replacement of HU with a JAK inhibitor) may also be warranted.

## Data Availability

No new data were created during this study.

## Abbreviations

The following abbreviations are used in this manuscript:

CRP	C-reactive protein
HU	hydroxyurea
JAK	Janus kinase
WBC	white blood cell count
